# Acoustic diagnosis of elastic properties of human tooth by 320 MHz scanning acoustic microscopy after radiotherapy treatment for head and neck cancer

**DOI:** 10.1186/s13014-020-01486-7

**Published:** 2020-02-17

**Authors:** Irem Demirkan, Gokhan Yaprak, Cemile Ceylan, Emriye Algul, Ceyda Ozcakir Tomruk, Bukem Bilen, Mehmet Burcin Unlu

**Affiliations:** 1 Department of Physics, Bogazici University, Bebek, 34342 Istanbul, Turkey; 2 Kartal Dr. Lutfi Kirdar Education and Research Hospital, Ministry of Health, 34890 Istanbul, Turkey; 3 Department of Biomedical Engineering, Kocaeli University, 41380 Kocaeli, Turkey; 4 Istanbul Oncology Hospital, 34846 Istanbul, Turkey; 5 Faculty of Dentistry, Department of Oral and Maxillofacial Surgery, University of Yeditepe, 34728 İstanbul, Turkey; 6 Global Station for Quantum Medical Science and Engineering, Global Institution for Collaborative Research and Education (GI-CoRE), Hokkaido University, Sapporo, 060-8648 Japan

**Keywords:** Scanning acoustic microscopy, Human teeth, Head and neck cancer, Radiotherapy, Microhardness

## Abstract

**Background:**

On the elastic profiles of human teeth after radiotherapy for head and neck cancers, generation of dental complications, which may bring several side effects preventing the quality of life, has not well clarified. Thus, we aimed to show the applicability of using 320 MHz Scanning Acoustic Microscopy (SAM) in the evaluation of the tooth damage acoustically at the micrometer level following radiation therapy, and also in the determination of the safe dose limits to impede severe dental damage.

**Methods:**

This prospective study was performed by SAM employed at 320 MHz by an azimuthal resolution of 4.7 μm resolving enamel and dentin. A total of 45 sound human third molar teeth collected between September 2018 and May 2019 were used for the acoustic impedance measurements pre- and post irradiation. Nine samples for each group (control, 2 Gy, 8 Gy, 20 Gy, 30 Gy and 60 Gy) were evaluated to acquire the acoustic images and perform a qualitative analysis. Scanning Electron Microscopy (SEM) images were obtained to establish a relationship between micromechanical and morphological characteristics of the teeth. Statistical analysis was conducted using the Student *t*-test succeded by Mann-Whitney U investigation (*p < .05*), while SEM images were assessed qualitatively.

**Results:**

The analysis included 45 sound teeth collected from men and women 18 to 50 years old. Post irradiation micromechanical variations of human teeth were significant only in the radiation groups of 30 Gy and 60 Gy compared to pre-irradiation group for enamel (7.24 ± 0.18 MRayl and 6.49 ± 028 MRayl; *p < 0.05*, respectively)*.* Besides, the teeth subjected to radiation doses of 20, 30 and 60 Gy represented significantly lower acoustic impedance values relative to non-irradiated group for dentin (6.52 ± 0.43 MRayl, 5.71 ± 0.66 MRayl and 4.82 ± 0.53 MRayl *p < 0.05*), respectively.

**Conclusions:**

These results are evidence for a safe acoustic examination device which may be a useful tool to visualize and follow the safe dose limits to impede severe dental damage through the radiation therapy treatment for head and neck cancers.

## Introduction

Head and neck cancers represent an inhomogeneous group of malignancies of the upper aerodigestive tract. This kind of cancer is seen at high frequencies of 500.000 new cases in a year [1]. Radiotherapy is prescribed to treat individuals with head and neck cancers. It is also used as a palliative together with chemotherapy drugs for malignancies, which are not suitably operated on. Radiotherapy can result in severe reactions of the oral cavity, though it aids healing and maintain the nature of tissues [2]. Individuals are principally treated by the radiation doses up to 60 Gy divided into 2 Gy fractioned daily doses, where the tissues of oral cavity, salivary glands and mandible are being irradiated [3]. According to the studies, individuals subjected to radiotherapy for head and neck cancers may encounter direct and indirect outcomes [4]. Alterations in salivary gland composition, candidiasis, vascular variations, oral mucositis and necrosis of soft tissues could originate during or in the first days or within months or years after the radiotherapy [4]. Apart from indirect effects, radiation is also known to directly damage the dental tissue to lose its structural identity through the reactions of variations in microhardness of enamel and dentin, elastic modulus, crystalline and dentin-enamel junction morphologies and matrix metalloproteinases (MMPs) [5–7]. Literature comprises a vast amount of investigations on histological, chemical, mechanical and morphological alterations on teeth subjected to ionizing radiation to gain knowledge for the causes of radiation therapy-induced effects [8–19]. Nevertheless, non-physiological conditions of these investigations extensively have limitations which may be harmful to the nature of the sample through exceeding the characteristics elastic limit [10]. However, the findings are still controversial, and thereby pathomechanism of complications in human teeth at microscale after radiation therapy is still ambiguous. In the last several years, researchers have focused on ultrasound for the inspection of hard tissues (i.e. teeth and bone) with the benefits of non-invasive, non-ionizing and non-harmful design [20–22]. In our recent proceeding study, we have also tested the applicability of SAM in tooth characterization after radiotherapy [23]. Current state of the art lacks diagnostic tools and has not well-developed. Thus, this study was motivated by the desire to suggest a hand-held tool which might enhance our understanding about micromechanicsm of the human tooth. It aims to show the applicability of 320 MHz Scanning Acoustic Microscopy (SAM) in the evaluation of the tooth alteration at the micrometer level following radiotherapy.

## Materials and methods

### Tissue selection and preperation

After review and approval of the proposed investigation through Ethical Committee of Kartal Doctor Lutfi Kirdar Education and Research Hospital, Ministry of Health, Istanbul with an approval number of 2018/514/18/9, human third molars of individuals 18 to 50 years old were collected by informed consent from Yeditepe University Faculty of Dentistry between September 2018 and May 2019. For this prospective study, of the initial extracted 65 human molar teeth, we included 45 (*n* = 45) teeth homologous to each other due to structural similarities. The established inclusion criterion was caries-free teeth. We excluded 20 teeth since they had cracks and fractures, presence of caries, appearance of metallic restorations, endodontic treatment. The extracted sound human teeth were stored in distilled water changed at 37 °C to mimic mouth moist. The roots and debris were removed. The teeth were bisected longitudinally by saw microtome (IsoMet 1000, Buehler) and then the surface of the remaining sections comprising enamel and dentin were polished (Phoenix Beta, Buehler) with a waterproof abbrasive paper of 1200 grit to attain a smooth surface to avoid superficial scattering of the sound waves. The thickness of the polished samples were approximately 1 mm. In our study, there is no requirement for the tooth sample to be thin sliced (i.e. 10 *μm*), stained and fixed for the visualization. The teeth were distributed into 6 groups (*n* = 9) in accordance with the irradiation doses: control, 2 Gy, 8 Gy, 20 Gy, 30 Gy and 60 Gy. The samples for each group were saved for further investigation in Scanning Electron Microscopy (SEM).

### Irradiation protocol

**Fig. 1:**
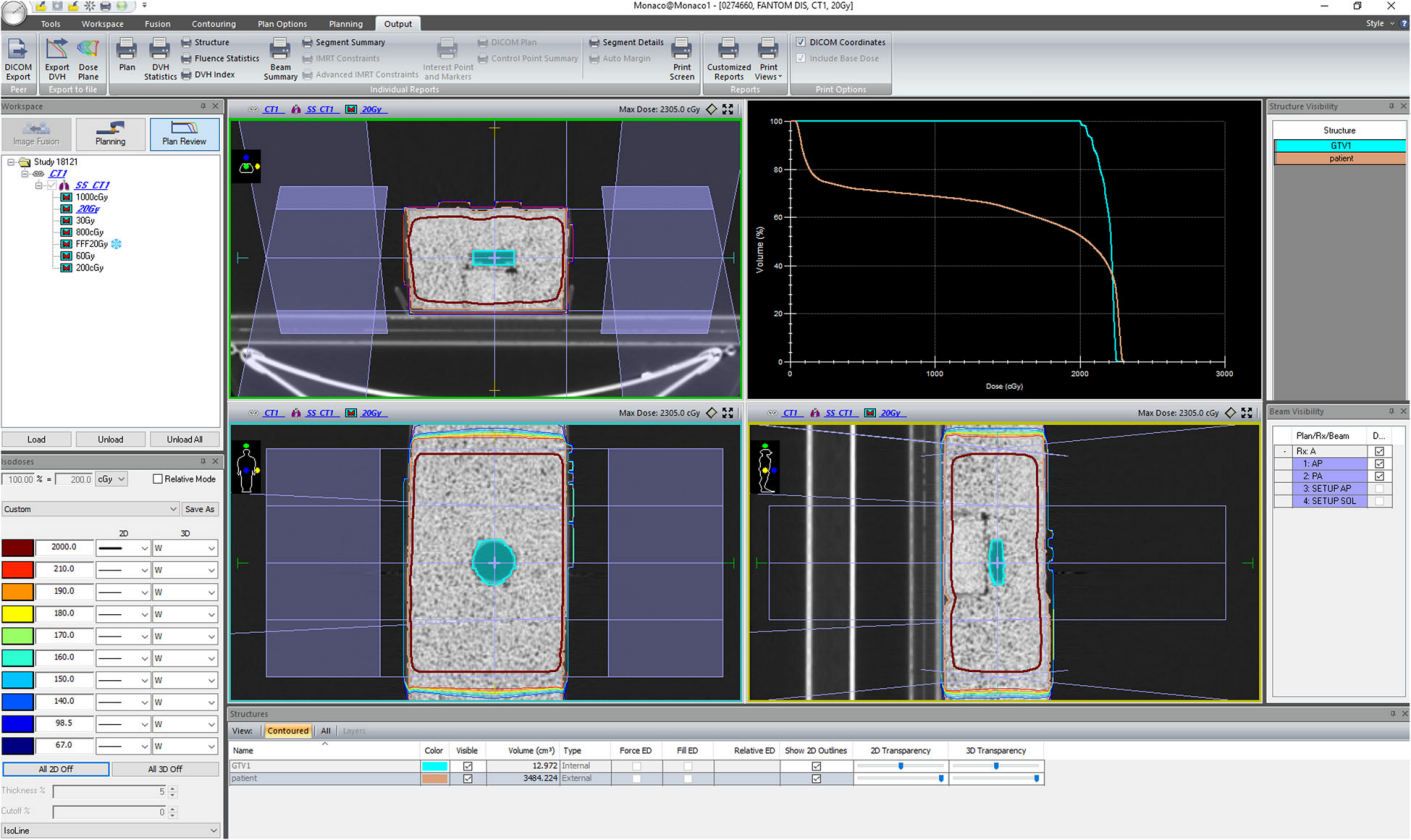
Details of the irradiation procedure for the polystyrene petri dish located within the rice phantom

Teeth samples were irradiated with 2 Gy, 8 Gy, 20 Gy, 30 Gy and 60 Gy doses in single fraction by a clinical linear accelerator (LINAC) (Elekta Versa HD, Elekta, Crawley, UK). 6 MV flat which has a dose rate of 600 cGy/min at dmax and unflattened photonbeams used which has a dose rate of 1200 cGy/min. All teeth were located in the 35 mm polystyrene petri dish. Irradiations were conducted with the positioned samples submerged in a custom-made rice phantom to achieve homogeneous dose distribution to the dishes as well as to simulate body. Before irradiation, the rice phantom was scanned through Computer Tomography (CT) with a slice thickness of 2 mm. The petri dish was deliniated as a Gross Tumor Volume (GTV). The dose distribution of the experimental design was acquired by Monaco Treatment Planning System. Figure 1 shows details of the irradiation procedure for the polystyrene petri dish which was located within the rice phantom.

The polystyrene petri dish was determined as a Gross Tumor Volume. The GTV was conformed 95/100 (95%) by prescribed doses using anterior-posterior/posterior-anterior (AP/PA) radiation fields, and Motor Unit (MU) calculation was done by collapsed cone algorithm by Treatment Planning System (Monaco 5.11.02v, TPS). Using 10 cm × 10 cm fields the number of monitor units were calculated by collapse cone calculation algorithm to consider heterogeneity to give the prescribed radiation doses that were 2, 8, 20, 30 and 60 Gy to the dental tissues located in the polystyrene petri dishes. Before irradiation of the dishes, output of LINAC was corrected to ensure dose differences at dmax smaller than 2% differences according to TRS 398 [24]. Irradiation doses of dishes were confirmed for each experiment setting through 0.6 cc farmer type ionization chamber. The position accuracy of the dishes within the phantom were checked for each irradiation through comparing Digital Reconstructed Radiography (DRR) images created by TPS and kilo-voltage (kV) images obtained by kV system of linear accelerator. Before each treatment the position control of the phantom using kV-kV images was controlled to ensure that the polystyrene petri dish was located within the same region with the tomograph planning.

### Scanning electron microscopy (SEM) analysis

For employing qualitative analysis by SEM (FEI-PHILIPS XL30-ESEM-FEG), a sample for each group in accordance with the radiation doses applied (control, 2 Gy, 8 Gy, 20 Gy, 30 Gy and 60 Gy) was chosen.

### Visualization of human teeth by scanning acoustic microscopy

For the measurements of acoustic characteristics of dental tissue before and after in vitro irradiation, we used 320-MHz SAM (AMS-50SI, HONDA ELECTRONICS CO., LTD, Toyohashi, Japan) in pulse-echo mode to directly measure sample reflection coefficient and, therefore acoustic impedance. The schematic of principle of SAM is given elsewhere [25]. Distilled water was applied as a couplant between transducer and the sample. A radio-frequency signal produced by way of a transmitter reaches to the piezoelectric transducer of the 320 MHz acoustic lens yielding sound waves. The sound waves move through a sapphire rod, and then are converted to spherical waves and focused on the surface of the sample by the help of an acoustic lens. Reflected acoustic waves by the sample are collected through the same acoustic lens and then turned to a radio-frequency signal by the transducer, and transmitted to a circuit of video-processor. The amount of reflected sound wave is directly comparable to the reflection coefficient:

1 $$ \mathrm{R}=\frac{Z_t-{Z}_w}{Z_t+{Z}_w} $$

in which Z_t_ and Z_w_ are the acoustic impedances of the teeth samples and the coupling agent water, respectively. The acoustic impedance (Z) is related to the elastic features of the sample and expressed as the product of mass density ρ and compressional wave c_p_ and commonly defined in MRayl (1 Rayl = 1 kg m^− 2^ s^− 1^). The acoustic impedance of the coupling liquid is used to confirm the calibration curve in the following equation [25]:

2 $$ \mathrm{Z}=\rho v={\left[\rho C\right]}^{1/2} $$

where *ρ* is the density, C is the elasticity coefficient, and v is the acoustic velocity of the sample under investigation. If the density of the embedding material is kept constant during the measurement, the variations in acoustic impedance will give the variation of tissue elasticity. The reflection coefficient is equivalent to the signal amplitude and establishes the brightness of the image. Higher acoustic impedance of the dental tissue sample accounts for a brighter image since the acoustic impedance of coupling liquid water is unchanged.

**Fig. 2:**
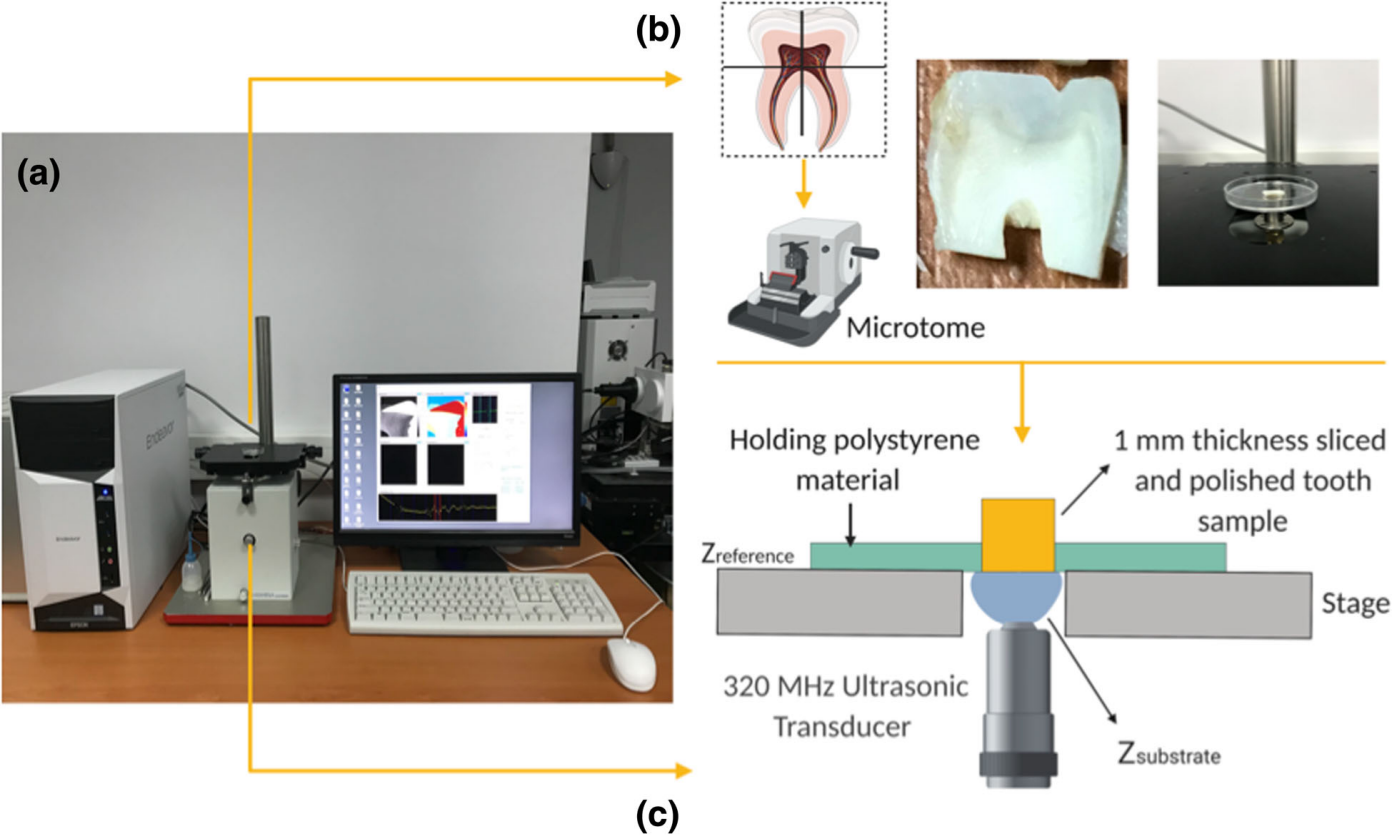
(**a**) Scanning Acoustic Microscopy (SAM) System (**b**) Schematic representation of tissue sectioning (cutting points from enamel and longutidunal direction) and localization for the measurement. (**c**) Schematic representation of acoustic impedance measurement mode. In this case, water is used as substrate. Tooth sample is not necessarily thin sliced. Just front surface must be polished to be very flat for the acoustic impedance measurement of the tooth sample

This study was conducted using specially constructed SAM for biological applications. It enables observation of the morphology and provides quantitative measures for biomechanical features of biological samples in the frequency range varying from 30 to 500 MHz**.** To visualize the samples, the microscope is equipped with a spherically focused broadband transducer with a central frequency 320 MHz since the azimuthal resolution has to be at least the same size of the sample for enamel prisms and dentin imaging [26]. The resolution is about 4.7 μm sufficient to visualize the microstructure details of dental tissue. The transducer includes a flat piezoelectric layer of ZnO with crystallographic orientation connected with a sapphire lens. From the bottom of the lens, its focal length was 0.50 mm with the spot size of 4 μm. The central frequency was 320 MHz, and acoustic pulse wave ranging from 200 to 400 MHz was focused on the junction between the tooth and the substrate, and passed through the substrate. The 320 MHz transducer was positioned under the sample on the X-Y stage where linear servo motors drove the both X-scan and Y-scan. Polystyrene petri dish holding the tooth sample was placed above the transducer as shown in Fig. 2 (created by BioRender), in which the front surface of the tooth was in contact with the transducer. Acoustic impedance of tooth was figured out using water as a substrate. The acoustic impedance of water as a substrate was 1.5 MRayl. Each tooth sample was irradiated and scanned along the X-Y axes in a frame with 300 × 300 pixels. Two-dimensional distribution of the acoustic impedance was obtained and the field of view was 4.8 mm × 4.8 mm with a 16 *μm* scan step. The treatment time for each tooth scan took 2–3 min. Eight pulse echo sequences were selected for each experiment to reduce noise.

### Statistical analysis

Statistical differences for acoustic impedance records were conducted through using Student *t*-test succeded by Mann-Whitney U analysis in GraphPad Prism 8 (GraphPad Software, San Diego, California) (*p < .05*), while SEM images were assessed qualitatively. All data were given as the average ± Standard Deviation (SD).

## Results

### Acoustic impedance imaging

**Fig. 3:**
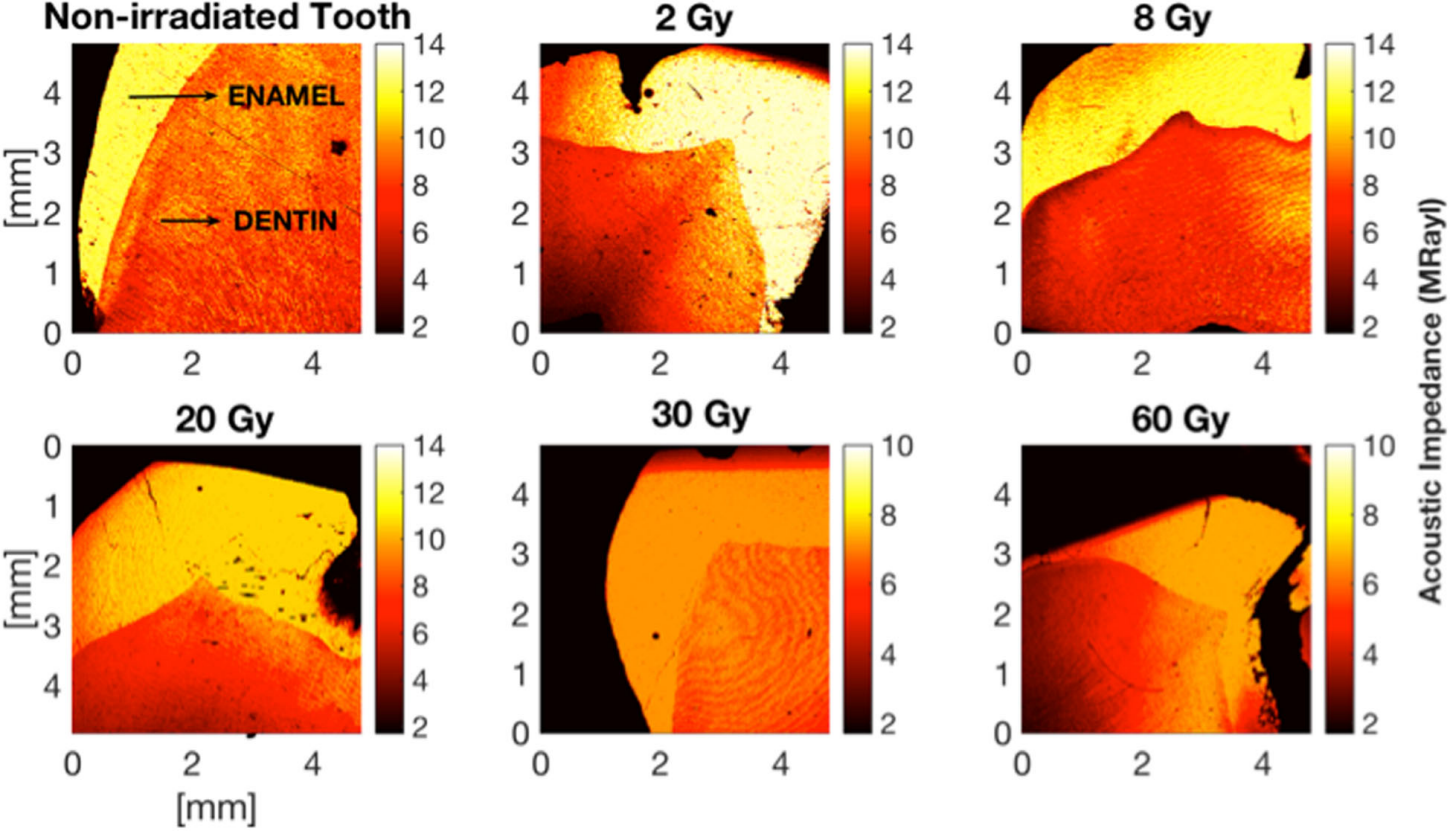
Two-dimensional acoustic impedance images of human tooth pre- and post-radiation therapy recorded by 320 MHz Scanning Acoustic Microscopy (SAM). The resolution was defined through the field of view of 4.8 mm × 4.8 mm for each image with a 300 × 300 scanning points with a scan size of 16 μm. All images were determined to visualize both enamel and dentin within a signal scan. Gradation in color bar (red to yellow) represent the variation after subjection to radiation doses which are clinically used to cure head and neck cancers. Sound dentin shows up higher acoustic impedance (orange to yellow) than that of the softened dentin through radiotherapy

Figure 3 displays non-uniform biomechanical features of dental tissue showing up alterations in the intensity of the reflected acoustic signal in bulk enamel and dentin for non-irradiated and for each radiation dose applied in this study. It can be seen that investigation at 320 MHz permitted to discern enamel and dentin as well as many microstructure details to be visualized in acoustic images. In the acoustic impedance image, SAM clearly monitored two-dimensional color distribution of the acoustic impedance of the caries-free human molars. The gradation color variations from control to 60 Gy two-dimensional images showed the course of the acoustic impedance values with respect to radiation doses. Enamel and dentin regions for non-irradiated group were composed of higher acoustic impedance values than those of the teeth of irradiated groups of 2 Gy, 8 Gy, 20 Gy, 30 Gy and 60 Gy. The softened dental tissue due to the ionizing radiation appeared in the image as bright orange areas of various tints relating to regions with a lower intensity of the acoustic signal.

**Fig. 4:**
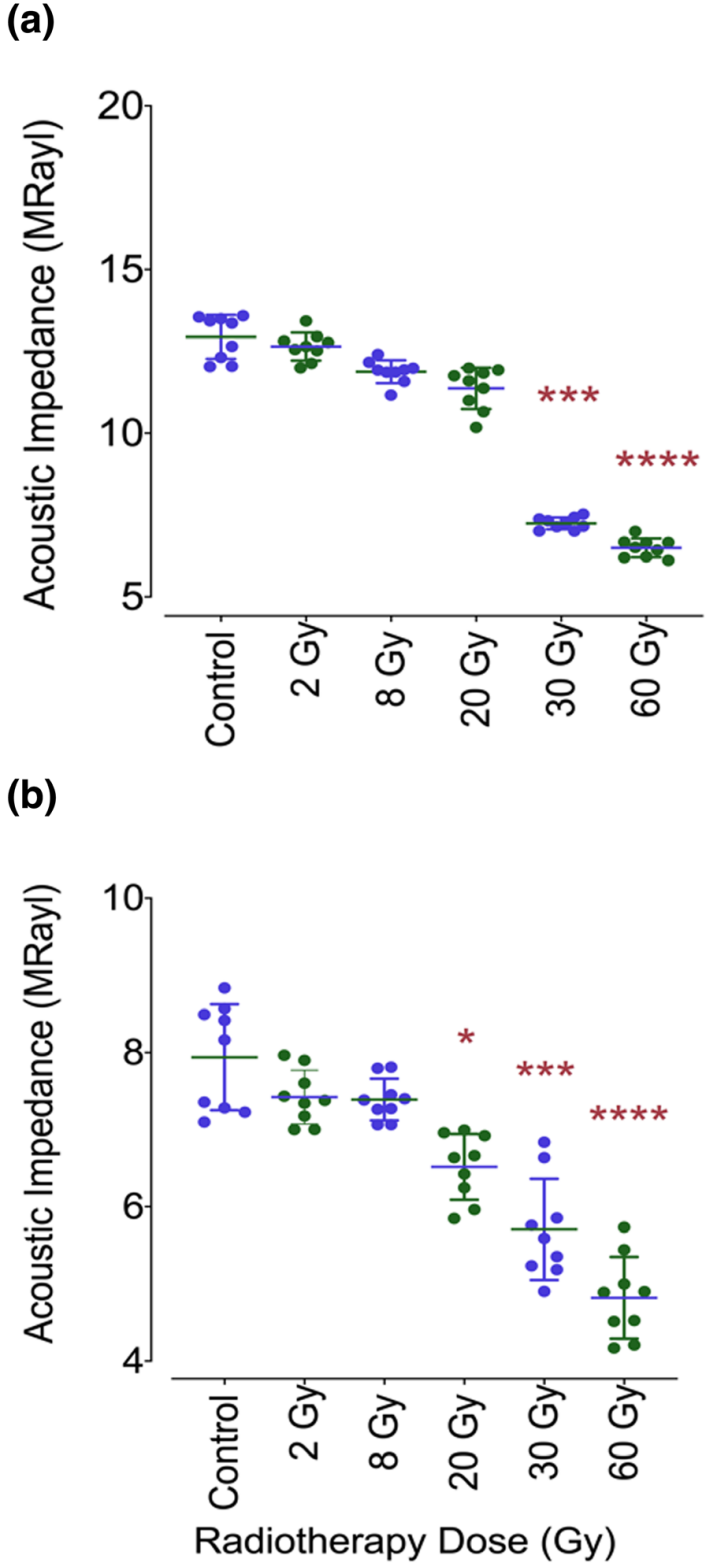
Acoustic impedance data distribution of non-irradiated and irradiated enamel and dentin as scatter plots. (**a**) shows acoustic impedance data for each group of enamel, and (**b**) shows acoustic impedance data for each group of dentin. Compared to no irradiation tooth group, the statistically significant irradiated groups were marked with the asterisk associating with * *p = .0296*, *** *p ≤ .0006*, **** *p < .0001*

For acoustic impedance analyses, in total, 45 sound human third molar were used. These samples were divided into groups, each of which contains 9 teeth, according to the radiation dose subjected. Table 1 and Fig. 4 demonstrate the acoustic impedance data distribution of non-irradiated and irradiated enamel and dentin.

**Table 1:** Mean and Standard Deviation of recorded acoustic impedance values for each region and irradiation dose obtained by 320 MHz Scanning Acoustic Microscopy (SAM). The statistically significant irradiated groups were marked with the asterisk ^*a*^

Dose	Acoustic Impedance Z (MRayl)					
	Enamel		*P* value	Dentin		*P* value
	Pre-irradiation	Post-irradiation		Pre-irradiation	Post-irradiation	
2 Gy (*n* = 9)	12.94 ± 0.67	12.65 ± 0.43	>.9999	7.94 ± 0.69	7.42 ± 0.35	> 0.9999
8 Gy (*n* = 9)		11.88 ± 0.35	.5594		7.39 ± 0.27	> 0.9999
20 Gy (*n* = 9)		11.37 ± 0.63	.0632		6.52 ± 0.43 ^a^	0.0296
30 Gy (*n* = 9)		7.24 ± 0.18 ^a^	.0002		5.71 ± 0.66 ^a^	0.0006
60 Gy (*n* = 9)		6.49 ± 0.28 ^a^	<.0001		4.82 ± 0.53 ^a^	< 0.0001

^a^Significant difference

Before irradiation of the samples, acoustic impedance of both enamel and dentin was measured. Enamel from sound teeth showed higher acoustic impedance value (Z = 12.94 ± 0.67 MRayl, *n* = 9) than that of dentin (7.94 ± 0.69 MRayl, *n* = 9), and therefore appeared to have higher elastic modulus due to the structure and organic components. The course of the acoustic impedance values with respect to radiation dose received can be clearly seen from Fig. 4. The teeth samples exhibited reduced values in enamel and dentin starting from radiation dose of 2 Gy to 60 Gy. The mean acoustic impedance value of enamel of the pre-radiation teeth group of 12.94 ± 0.67 MRayl was the highest among the groups. The acoustic impedance values compared to non-radiated control group displayed significant variations after accumulated radiation doses of 30 Gy and 60 Gy, while irradiation doses lower than 30 Gy showed no significant effect on acoustic impedance distribution of the enamel (Fig. 4). For enamel, after applying 30 Gy in vitro irradiation, the acoustic impedance was measured 7.24 ± 0.18 MRayl, which was lower than the acoustic impedance value of pre-irradiation group of 12.94 ± 0.67 MRayl. The difference from the control group was found statistically significant (*p* = .*0002*). After 60 Gy radiation dose application, the mean acoustic impedance value of 6.49 ± 028 MRayl was measured, which was statistically lower than that of the non-irradiated teeth group (*p* < *.0001*). Lower mean acoustic impedance values than that of the non-irradiated teeth group were also observed after 2 Gy, 8 Gy and 20 Gy in vitro irradiation which were figured out 12.65 ± 0.43 MRayl, 11.88 ± 0.35 MRayl, 11.37 ± 0.63 MRayl, respectively (*p* > *.05*).

For dentin, a trend of varying acoustic impedance values was also obtained as the teeth affected by radiation dose received. The average acoustic impedance value of 9 sound dentin, which were not irradited, was higher than those of radiated dentin (7.94 ± 0.69 MRayl). Under radiotherapy doses of 2 Gy and 8 Gy, irradiation obviously had a minor effect on the biomechanical characteristic of the dentin. The recorded acoustic impedance values for 2 Gy and 8 Gy were 7.42 ± 0.35 MRayl and 7.39 ± 0.27 MRayl, respectively. These differences were not reported as significant. Compared to control group, the effect of in vitro subjection of the radiotherapy dose of 20 Gy towards the teeth was measured as the acoustic impedance of the dentin diminished from the mean acoustic impedance value of 7.94 ± 0.69 MRayl to 6.52 ± 0.43 MRayl. The variation in the acoustic impedance was stated as a significant difference at the *p* = .0296 level. The comparison between 30 Gy radiation dose group and the pre-irradiated group revealed a significant change from 7.94 ± 0.69 MRayl for the sound dentin group to 5.71 ± 0.66 MRayl for post-irradiation teeth group (*p* = .0006). For dentin, 60 Gy irradiation had the highest level of influence on the human teeth in terms of difference in mean acoustic impedance value. Non-irradiated group had the mean acoustic impedance of 7.94 ± 0.69 MRayl on the other hand, 60 Gy radiation dose received group had the average acoustic impedance of 4.82 ± 0.53 MRayl exhibiting a significany change (*p* < .*0001*).

### Scanning electron microscopy

**Fig. 5:**
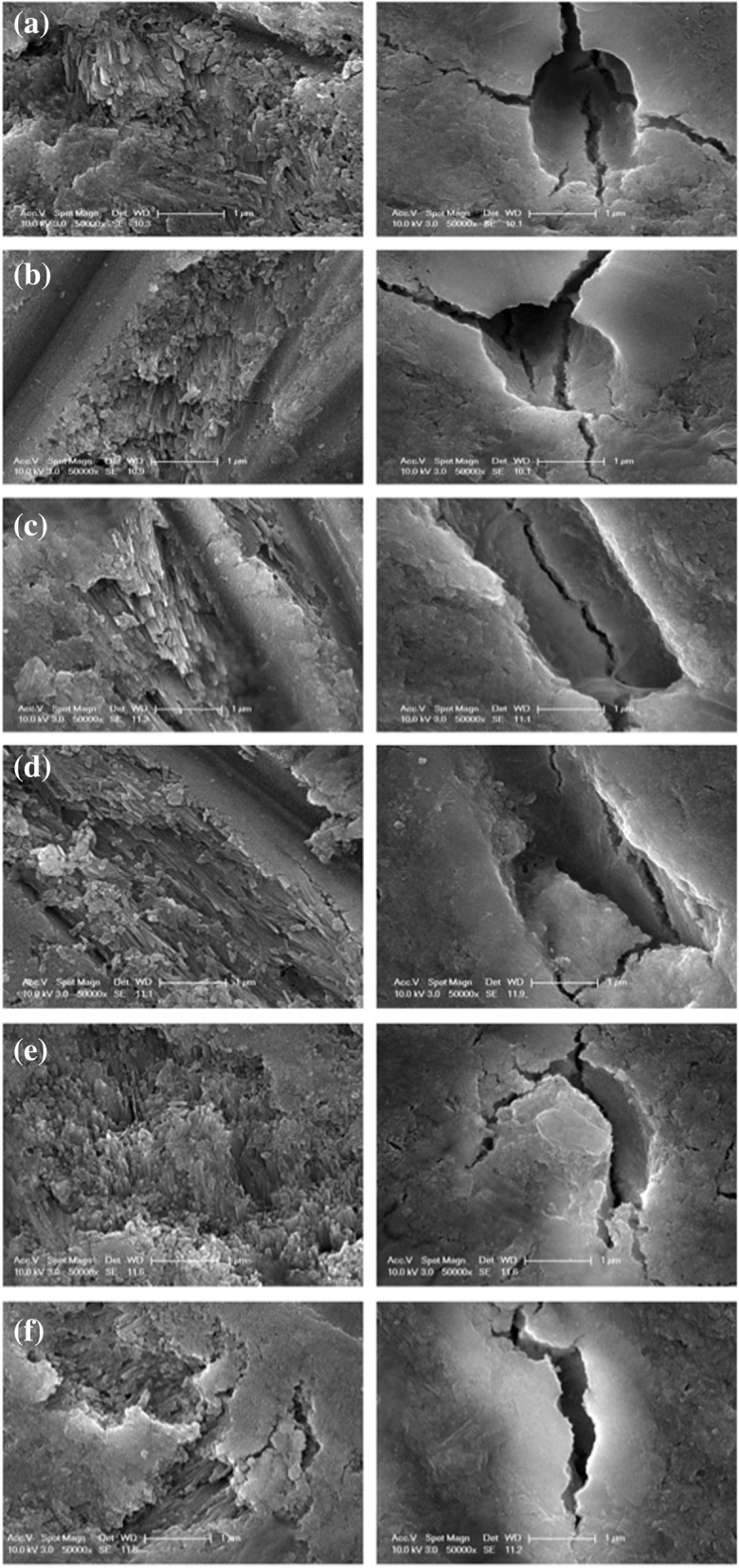
Electron micrographs of the enamel and dentin of the human molar teeth. The images were obtained by Scanning Electron Microscopy (SEM) at 50000x. Left-hand side images represent enamel, while right-hand side show dentin. (**a**) Control (**b**) 2 Gy, (**c**) 8 Gy, (**d**) 20 Gy, (**e**) 30 Gy and (**f**) 60 Gy, respectively

The enamel of pre-irradiation teeth showed the presence of regular normal enamel at all depths with the well-organized prisms as it can be clearly seen in Fig. 5. Similar characteristics were also observed after subjection of radiation doses of 2 Gy, 8 Gy and 20 Gy on enamel surface. On the other hand, following exposure to 30 Gy, a slight micro-morphological variation was visualized in the prismatic structure of the enamel with all of the inspected regions of the treated tooth (Fig. 5). With an increasing dose of radiation to 60 Gy, micro-morphological variations appeared more evident. Exposure to 60 Gy hindered the observation of interprismatic structure and hydroxypatite crystals making the enamel surface amorphous.

As for dentin, the pre-irradiation group demonstrated well-defined intertubular, peritubular and dentinal tubule together with an normally ordered collagen network. The SEM photomicrographs of the groups subjected to radiation doses of 2 Gy and 8 Gy showed similar micromorpholocial properties. In comparison to sound dentin images, treatment of the teeth by 30 Gy and 60 Gy radiation doses caused a progressive variation in the morphology in all of the assessed parts. Due to the increasing radiation doses, intertubular dentin, peritubular dentin and dentinal tubular illustrated unregular structure compared to non-irradiated group. The dentinal tubules started disappearing for the 60 Gy irraditation 50000x magnification.

## Discussion

Though the radiotherapy efficaciously cures individuals with head and neck cancers, it may bring several side effects lowering the quality of life. The deterioration of the elasticity of the human tooth after radiotherapy has not well developed. Accordingly, this study was motivated by the desire to suggest a hand-held tool which might enhance our understanding about the micromechanical properties of the human tooth. Our aim was to show the applicability of 320 MHz Scanning Acoustic Microscopy (SAM) in the evaluation of the tooth alteration at the micrometer level following radiation therapy. The radiation dose dependence of the acoustic impedance variations on the extracted human teeth was quantitatively identified using 320 MHz SAM in the acoustic impedance mode. The outcomes suggest that SAM is a promising tool to analyze the elasticity and its distribution in the targeted tissue in situ. Based upon the principle of acoustic impedance measurement, a hand-held acoustic probe, which establishes a relationship between acoustic impedance variations and the radiation doses, may be designed in future. Hence, acoustic impedance measurements in situ could pave the way to follow dental tissue at the microscale resolution to guide and ensure the safety of individuals treated by radiotherapy with a non-invasive and non-ionizing approach in clinical settings.

Findings in the proposed study showed that the average acoustic impedance values of non-irradiated sound dentin were lower than those of non-irradiated sound enamel. This difference was as anticipated, since enamel differs from dentin in the form of structure and orientation. A link between structural variations and elastic features of enamel and dentin was formely documented through determination of elastic modulus [27]. Results imply that SAM could be used to distinguish enamel from dentin through the acoustic impedance quantification.

The study further demonstrated that enamel behaved rather differently from dentin as a function of the irradiation dose. The findings of the method gave that ionizing radiation resulted in a dose-dependent reduction in the enamel microhardness for cumulative doses of 30 Gy and 60 Gy. For the doses, a conformable relationship between the variations in acoustic impedance and prismatic structure of enamel was established. SEM images showing the prismatic structure of enamel appeared more evident with the radiation doses of 30 Gy and 60 Gy, which matched the literature [28]. This observation was also in agreement with the previous study of teeth indicated that microhardness of irradiated human teeth was lower compared to non-irradiated human teeth groups [16]. Radiotherapy can lead to restructuring of the crystal structures of mineralized tissues, and accordingly makes changes in the structural elasticity. The enamel is organized by prisms, and the structural orientation defines the non-isotropic appearance of the enamel and reflects it mechanical features [29]. SEM investigations for 30 Gy and 60 Gy irradiation doses unclosed morphological variations in the enamel marked through progressively disorganized interprismatic structure, as previously studied [29]. The elasticity and the integrity of the teeth were affected, and the radiogenic damage was observed as a lower microhardness in enamel proved by acoustic impedance. This may occur because of decarboxylation of the tissue [6]. The lower acoustic impedance value and the change in enamel crystalline structure might be the reason for the increased risk of dental deterioration after radiotherapy. The lack of statistical differences in the acoustic impedance after 2 Gy, 8 Gy and 20 Gy dose exposures has to be clarified, as the enamel mechanical structure and composition might be affected through ionizing radiation; yet those modifications may be incipient evidences of slight micro-morphological alterations in the enamel*.* This result should be regarded for the establishment of protocols to hinder or weaken deleterious influences during radiotherapy. The elastic properties of dentin undergone radiotherapy appeared to be much more than those of enamel. Following cumulative irradiation with 20 Gy, decreasing trend in acoustic impedance values of dentin was observed. As for SEM images, it was observed irradiated teeth by cumulative radiation doses of 20 Gy, 30 Gy and 60 Gy presented an increasing morphological deterioration. Starting with 20 Gy, presence of fissures, cracks and obliteration in dentinal structure became notable. The reduction in dentin elasticity evidenced by the acoustic impedance measurements may be expressed by the variations in organic components in dentin tubules .

The literature has also reported ionizing radiation may lead to reduction in microhardness [28]. Enamel and dentin responded differently to the radiotherapy, which is possibly due to the water content. Dentin has higher water content equal to 10% whereas enamel has 4% by weight [30]. Resulting from the physical interaction of ionizing radiation in water, radiotherapy causes production of free radicals and hydrogen peroxide [31]. Thereby, in terms of water content, dentin may be more unstable and susceptible to the radiotherapy, and a substantial influence on the microhardness of dentin structure was observed. Acoustic impedance of dentin was progressively reduced with the increasing radiation doses. SEM images reflecting micro-morphological alterations such as obliteration and fissures in dentinal structure may also confirm the progressive differences in elasticity of dentin. The proposed study was employed in vitro, therefore it has constrains on the reproduction factors (i.e., alterations in oral microflora and hyposalivation) that cannot be taken into account.

## Conclusions

Determination of acoustic impedance values made a reliable technique for quantification of microhardness characteristics of dental tissues with no mechanical harm to the samples. Our findings support the evidence of a direct radiogenic-related influence on enamel and dentin at the micrometer level. Scanning Acoustic Microscopy (SAM) was suggested as a feasible method which enables detection of alterations in the microhardness due to the radiation therapy through the acoustic impedance values of enamel and dentin. As a future study, we plan on applying a dose of 2Gy/fraction up to a cumulative dose of 60 Gy. The measurements of post-irradiation enamel and dentin acoustic impedance are aimed to be measured after every 2 Gy of irradiation till completing 30 irradiation cycles. Based upon the principle of acoustic impedance measurement, a hand-held ultrasonic stiffness checker establishing a relationship between acoustic impedance variations and the radiation doses may be designed. The link between micro-mechanical to morphological variations needs further assessments.

## Data Availability

Please contact the corresponding author for data requests.

## Abbreviations

AP/PA: Anterior-posterior / Posterior-anterior
CT: Computer Tomography
GTV: Gross Tumor Volume
LINAC: Linear Accelarator
SAM: Scanning Acoustic Microscopy
SEM: Scanning Electron Microscopy
